# The evolutionary drivers of primate scleral coloration

**DOI:** 10.1038/s41598-022-18275-9

**Published:** 2022-08-18

**Authors:** Alex S. Mearing, Judith M. Burkart, Jacob Dunn, Sally E. Street, Kathelijne Koops

**Affiliations:** 1 Department of Archaeology, Fitzwilliam Street, University of Cambridge, Cambridge, CB2 1QH UK; 2 Department of Anthropology, University of Zurich, 8057 Zurich, Switzerland; 3 School of Life Sciences, Anglia Ruskin University, Cambridge, CB1 1PT UK; 4 Department of Cognitive Biology, University of Vienna, 1090 Vienna, Austria; 5 Department of Anthropology, University of Durham, Durham, DH1 3LE UK

**Keywords:** Biological anthropology, Animal behaviour

## Abstract

The drivers of divergent scleral morphologies in primates are currently unclear, though white sclerae are often assumed to underlie human hyper-cooperative behaviours. Humans are unusual in possessing depigmented sclerae whereas many other extant primates, including the closely-related chimpanzee, possess dark scleral pigment. Here, we use phylogenetic generalized least squares (PGLS) analyses with previously generated species-level scores of proactive prosociality, social tolerance (both n = 15 primate species), and conspecific lethal aggression (n = 108 primate species) to provide the first quantitative, comparative test of three existing hypotheses. The ‘self-domestication’ and ‘cooperative eye’ explanations predict white sclerae to be associated with cooperative, rather than competitive, environments. The ‘gaze camouflage’ hypothesis predicts that dark scleral pigment functions as gaze direction camouflage in competitive social environments. Notably, the experimental evidence that non-human primates draw social information from conspecific eye movements is unclear, with the latter two hypotheses having recently been challenged. Here, we show that white sclerae in primates are associated with increased cooperative behaviours whereas dark sclerae are associated with reduced cooperative behaviours and increased conspecific lethal violence. These results are consistent with all three hypotheses of scleral evolution, suggesting that primate scleral morphologies evolve in relation to variation in social environment.

## Introduction

**Figure 1:**
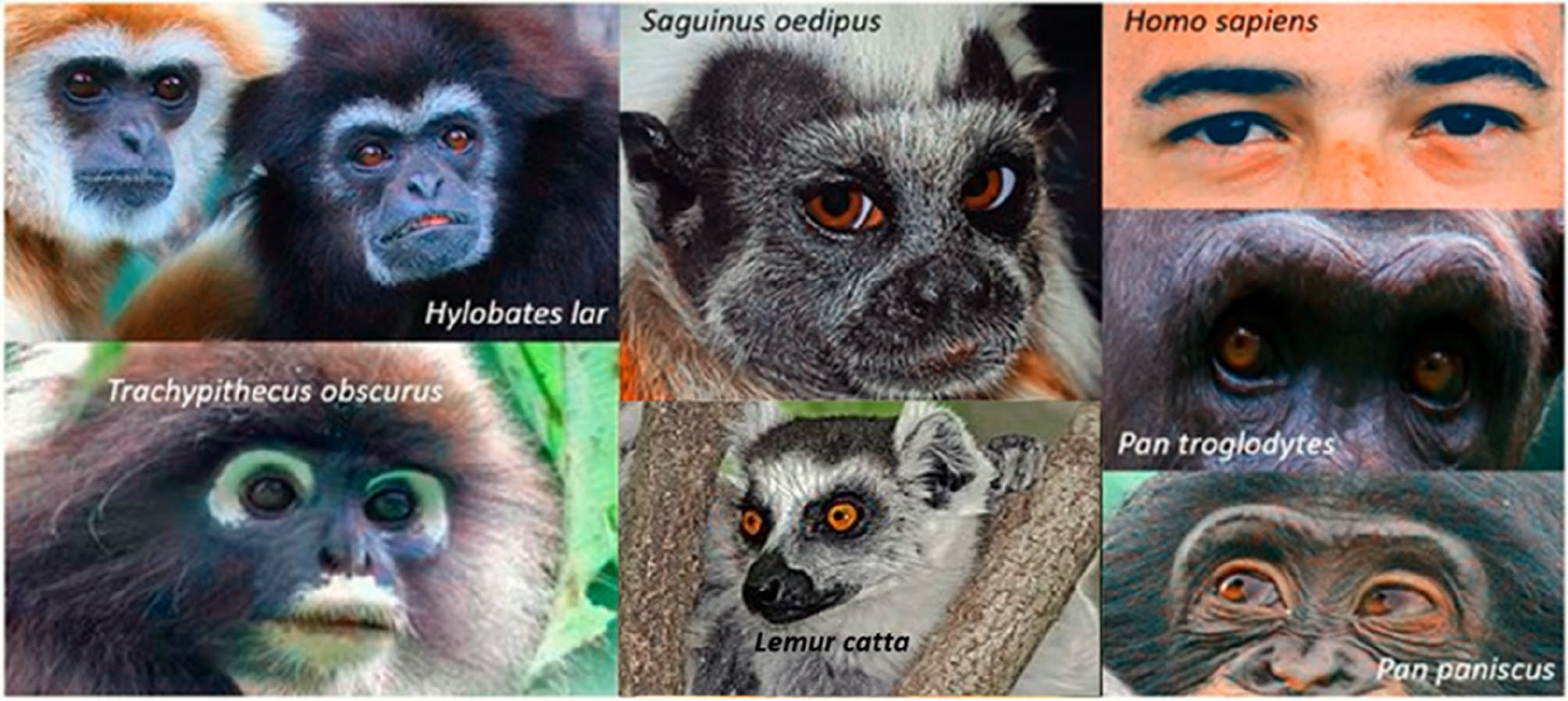
Ocular diversity in the primate order. H. lar photo under creative commons usage, credit to user: MatthiasKabel (https://commons.wikimedia.org/wiki/Hylobatidae#/media/File:Hylobates_lar_pair_of_white_and_black_02.jpg). T. obscurus photo under creative commons usage, credit to Lip Kee Yap (https://commons.wikimedia.org/wiki/File:Trachypithecus_obscurus.jpg). S. oedipus photo under creative commons usage, credit to Michael Gäbler (https://en.m.wikipedia.org/wiki/File:Saguinus_oedipus_(Linnaeus,_1758).jpg). L. catta photo under creative commons usage, credit to Charles J. Sharp (https://commons.wikimedia.org/wiki/File:Ring-tailed_lemur_(Lemur_catta)_in_tree.jpg). H. sapiens photo is in the public domain, credit to Fernanda Latronica (https://www.pexels.com/photo/close-up-photography-of-bearded-man-713520/). P. troglodytes photo provided by the Royal Burgers’ Zoo, Netherlands. P. paniscus photo is under creative commons usage, credit to William H. Calvin (https://commons.wikimedia.org/wiki/File:Bonobos_11yr_male_3yr_male_grin_Twycross.jpg).

The primate order contains a remarkable amount of variation in external ocular morphology (Fig. 1), including differences in scleral volume, width-height ratios and pigment profiles^1–8^. Scleral volumes and width-height ratios have been linked in phylogenetic comparative analyses to social (i.e., group size and neocortex ratio), ecological (i.e., habitat use) and life history (i.e., body mass) drivers^3^. However, no phylogenetic comparative study to our knowledge has yet examined the evolutionary drivers of scleral pigment across primate species. Although, for simplicity, we refer to “scleral” pigmentation throughout, the sclera is covered by a thin conjunctival membrane. Whether it is the sclera, the conjunctiva, or both that may be pigmented is not known for each species of non-human primate studied^2,3,5,6,8^.

Within the primate order, humans are often considered to uniquely possess depigmented sclerae whereas most non-human primate species, including the closely related chimpanzee, instead synthesise dark scleral pigment^1,2,8,9^. Interestingly, the equally closely-related bonobo possesses sclerae of an intermediate average brightness between humans and chimpanzees^5,6,10^. One hypothesis for the evolution of divergent scleral morphologies is that white sclerae may be pigment-related by-products associated with self-domestication processes^11^. The ‘neural crest cell hypothesis’ of domestication predicts that selection for tolerance and against aggression results in reduction in the number and migration velocity of neural crest cells in early embryogenesis. This alteration may be responsible for the domestication-syndrome, a range of behavioural and morphological co-emergents with docility^12,13^. Pigment-producing melanocytes are derived from neural crest cells, so the reduction of melanocytes in a pale sclera is potentially explicable as a correlated by-product of selection for social tolerance^11,14^. However, the precise relationship between tolerance, aggression and domestication between species is currently poorly understood and to-date, this topic remains largely under-explored by comparative investigations across mammal clades^15^.

Alternative hypotheses rely on non-human primate eyes being communicative—a matter of considerable debate^3,6,16^. The cooperative eye hypothesis^9^ suggests that, in human evolution, the human depigmented sclera has functioned to facilitate hyper-cooperative behaviours observed^9,17^ through, for example, the establishment of joint attentional states. A white background may signal iridal direction more conspicuously and in a manner that is more difficult to conceal than would a dark background^7,10,18^. Contrastingly, the ‘gaze camouflage hypothesis’ argues that the presence of dark scleral pigment in many primate species functions as iridal direction camouflage in environments of high competition and/or predation^2^. As evidence, authors note the metabolic cost required to synthesise pigment which may indicate an adaptive function^2^. No function of scleral pigment has yet, to our knowledge, been comparatively determined, though it has been suggested that ocular pigment may instead provide protection against UV radiation^19,20^.

However, the degree to which non-human primates can respond to conspecific ‘glance’ cues, i.e., movements of the eyes independently of the head rather than ‘gaze’ cues, i.e., movements of the head is unclear. Experimental evidence that non-human primates can interpret referential information from glance cues is sparse^6^, though studies are few and have examined only several species to our knowledge. By contrast, several phylogenetically diverse species of non-human primates are reported to be able to follow head direction^21–24^ and may be able to infer intentionality from this cue. For example, chimpanzees have been observed to avert their head direction from a high value food item if they, alone, are knowledgeable about its location and are in the presence of a dominant conspecific^25^.

Importantly, chimpanzees are able to move their eyes independently of their head similarly to humans^26^ and have been shown to follow others’ iridal direction at ecologically relevant distances^26,27^, though typically do not utilise glance cues to ascertain referential information in object-choice tasks^16,28–32^. Similarly, rhesus macaques (*Macaca mulatta*) have been shown to be reflexively sensitive to glance stimuli^33^, though it is not known whether they can derive referential information from this, and capuchins (*Sapajus apella*)^34^ also do not appear able to discern referential cues from glance stimuli.

Recently, a number of studies have examined primate gaze conspicuousness by measuring the brightness contrast between the sclera and iris. Creating a contrast ratio, Perea-Garcia et al.^5^ found that humans and *Pan* possessed comparable levels of gaze conspicuousness, though this method was reported as being statistically biased towards comparable results^6,8^. Instead, measuring the iridoscleral brightness range, Caspar et al.^6^ do not replicate this result, but find conspicuousness overlap between humans, gorillas (*Gorilla gorilla*) and Sumatran orang-utans (*Pongo abelii*). The authors take this as evidence against divergent eye morphologies being communicative. They add that, although all studied primates do display visible sclera^3^, some primate species display much less than others, presumably reducing the efficacy of sclera as a potential social cue in these cases.

Here, we utilise the remarkable diversity of primate ocular morphologies to provide the first quantitative, comparative analysis of primate scleral colouration. We compare scleral brightness with three behavioural measures: proactive prosociality, social tolerance, and conspecific lethal violence. Prosociality refers to any behaviour which benefits another organism irrespective of any benefit to themselves^35^. Prosociality can be reactive, e.g., in response to help-calls or subtle coercion, or proactive, that is, unsolicited^36^. Social tolerance, that is, the tolerance towards groupmates, is not prosocial, but equally is also non-competitive. Competitive behaviours, such as conspecific lethal violence, are often similarly adaptive social strategies^36^. These three measures enable the examination of the evolutionary role of scleral pigment across different social dynamics. Conspecific lethal violence is indicative of highly competitive social environments, proactive prosociality of highly cooperative environments and social tolerance represents a behaviour of intermediate social value.

The application of the gaze camouflage hypothesis would predict heightened lethal aggression to be associated with darker sclerae, and for dark sclerae to be likewise associated with low values of proactive prosociality and social tolerance. Both the ‘cooperative eye hypothesis’ (directional selection) and the ‘self-domestication hypothesis’ (correlated by-product) similarly predict white sclerae to be associated with heightened proactive prosociality, although the self-domestication hypothesis explains the presence of prosociality as a correlated by-product of selection for social tolerance^13,17^. Hence, an association between scleral brightness and proactive prosociality, but not social tolerance, could be taken as evidence against the self-domestication perspective.

## Results

### Proactive prosociality

**Figure 2:**
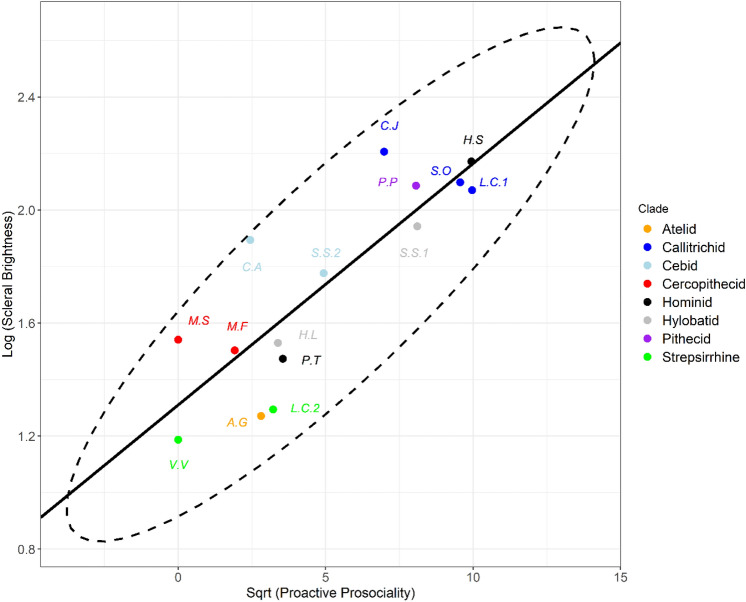
Proactive prosociality and scleral brightness. PGLS regression plot comparing log (proactive prosociality) with log (scleral brightness) in 15 primate species. Ellipse showing bivariate outliers to a 95% confidence interval. *CJ* Callithrix jacchus, *HS* Homo sapiens, *SO* Saguinus oedipus, *LC1* Leontopithecus chrysomelas, *PP* Pithecia pithecia, *SS1* Symphalangus syndactylus, *SS2* Saimiri sciureus, *CA* Cebus apella, *MS* Macaca silenus, *MF* Macaca fuscata, *HL* Hylobates lar, *PT* Pan troglodytes, *LC2* Lemur catta, *AG* Ateles geoffroyi, *VV* Varecia variegata.

Log (Scleral brightness) was significantly positively associated with sqrt (proactive prosociality) scores across the 15 primate species. Where lambda was taken at its maximum likelihood (λ_ML_ = 0), a statistically significant relationship was observed (p =  < 0.001, R^2^ = 0.711, estimate = 0.085, t = 5.66; Fig. 2). Similarly, where lambda was assumed to equal 1, a highly conservative comparison, the statistically significant relationship remained (p =  < 0.001, R^2^ = 0.723, estimate = 0.083, t = 5.83).

### Social tolerance

**Figure 3:**
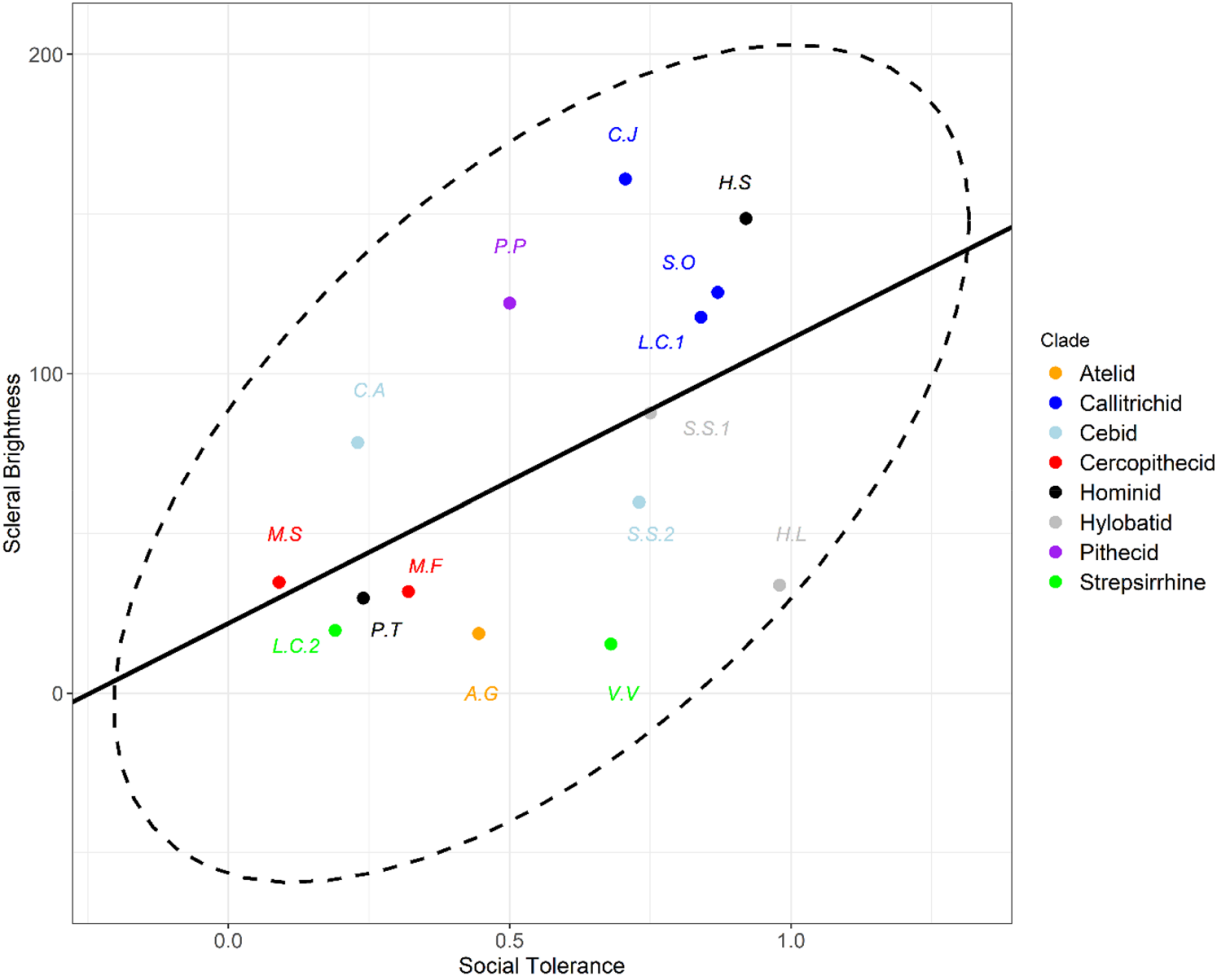
Social tolerance and scleral brightness. PGLS regression plot comparing social tolerance scores with scleral brightness in 15 primate species. Ellipse showing bivariate outliers to a 95% confidence interval. *CJ* Callithrix jacchus, *HS* Homo sapiens, *SO* Saguinus oedipus, *LC1* Leontopithecus chrysomelas, *PP* Pithecia pithecia, *SS1* Symphalangus syndactylus, *SS2* Saimiri sciureus, *CA* Cebus apella, *MS* Macaca silenus, *MF* Macaca fuscata, *HL* Hylobates lar, *PT* Pan troglodytes, *LC2* Lemur catta, *AG* Ateles geoffroyi, *VV* Varecia variegata.

Scleral brightness was significantly positively associated with social tolerance scores across the 15 primate species. Where lambda equals its maximum likelihood (λ_ML_ = 0), a significant relationship was observed (p = 0.047, R^2^ = 0.269, estimate = 90.58, t = 2.19; Fig. 3). Likewise, where weighted by lambda = 1, the significant association remains (p = 0.03, R^2^ = 0.314, estimate = 95.57, t = 2.44).

### Conspecific lethal aggression

**Figure 4:**
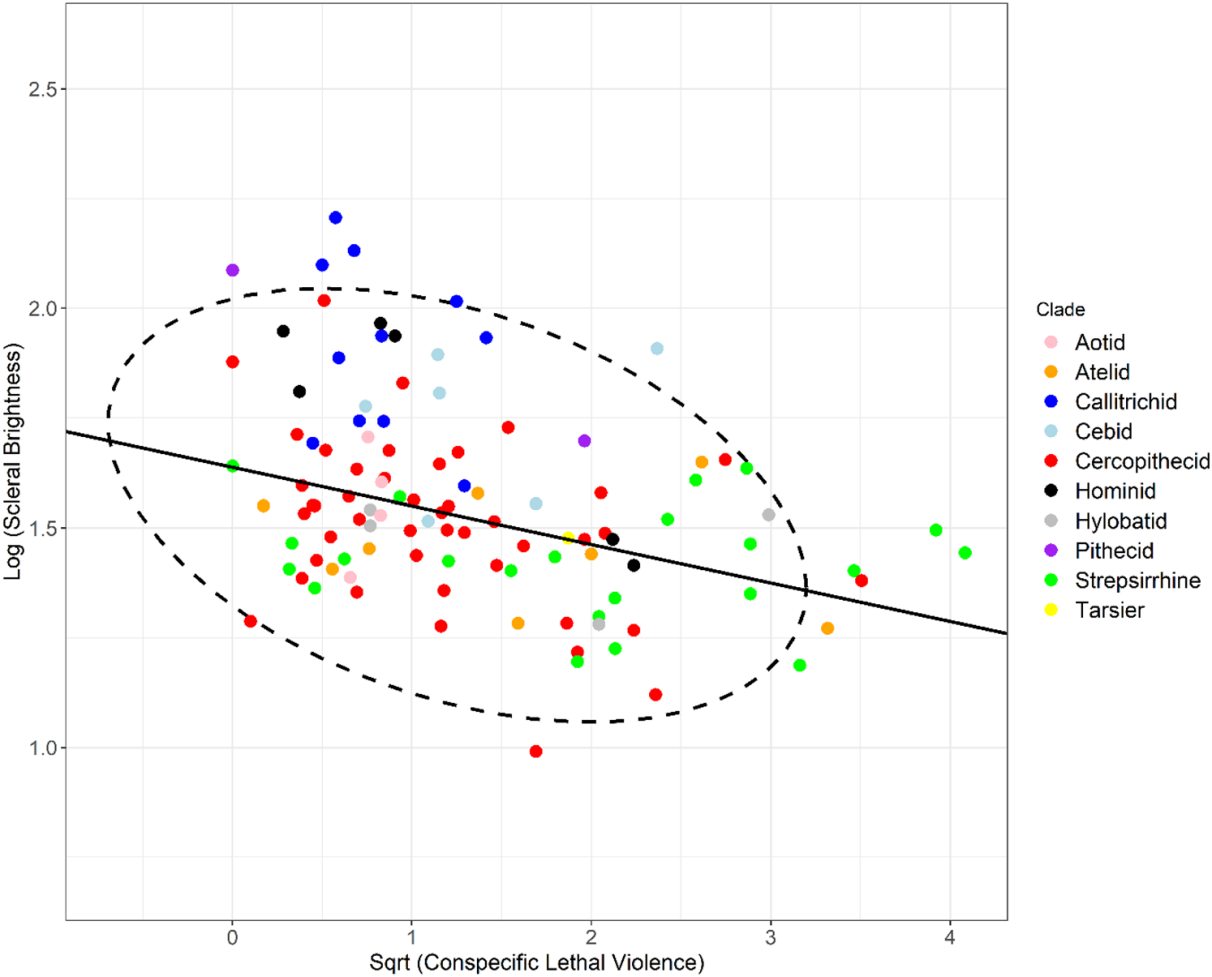
Conspecific Lethal Violence and scleral brightness. PGLS regression plot comparing the percentage of deaths due to conspecific lethal violence with log (scleral brightness) in 108 primate species. Ellipse showing bivariate outliers to a 95% confidence interval.

A significant negative association was observed across the 108 primate species between log (scleral brightness) and sqrt (conspecific lethal violence). Where lambda was taken at its maximum likelihood (λ_ML_ = 0.694), a statistically significant observation was observed (p =  < 0.001, R^2^ = 0.119, estimate = − 0.088, t = − 3.791; Fig. 4). Similarly, where lambda was assumed to equal 1, a statistically significantly relationship remains (p =  < 0.001, R^2^ = 0.307, estimate = − 0.144, t = − 6.85).

## Discussion

Our findings are consistent with all three hypotheses tested: the self-domestication, cooperative eye and gaze camouflage hypotheses. We show that scleral pigmentation varies with differences in social behaviours between primate species. Proactive prosociality, an experimentally derived measure of the degree to which individuals helped their groupmates with no possibility of directly benefitting themselves, was associated with significantly increased scleral brightness. Social tolerance, a measure of the evenness of the distribution of food items in the same species, likewise showed a significant association with scleral brightness. The larger-scale (n = 108) comparison between scleral brightness and conspecific lethal violence also returned a consistent result. Scleral brightness was significantly negatively associated with the percentage of deaths attributable to conspecific lethal aggression.

The presence of scleral pigmentation is sometimes argued to be a functional adaptation, rather than a product of random drift, due to the metabolic cost incurred in synthesising dark pigment^2^. The finding that the extent of conspecific lethal violence negatively predicts scleral brightness (i.e., predicts darker, more pigmented sclerae), may indicate that scleral pigment can function as a mechanism of gaze camouflage^2^. Chimpanzees, who are considered more reactively aggressive and less cooperative than humans^11,13^, and who likewise possess darker sclerae^5^, have been shown to use gaze aversion^25^ when engaged in food competition. The presence of pigment could confer an advantage in terms of concealing gaze direction from groupmates in a competitive context^2^. However, many primate species do not possess large volumes of visible sclera and sometimes do possess bright irises which may contrast the pupil^5,6^, calling into question the degree to which comparatively small volumes of pigmented sclerae could camouflage gaze and, subsequently, intentionality. In addition, the degree to which eye direction, independent of head direction, can be interpreted as a social cue in non-human primates is unclear, sparsely researched in most species and has been challenged^6^. Instead, scleral pigment may represent an alternative function such as photo-regulation^19,20^ with exceptions delineating in line with the strength of self-domestication processes^11^.

Additionally, increased scleral brightness, i.e., depigmentation, could also be potentially functional. Our results indicate that increased scleral brightness positively predicts increased cooperative behaviours and reduced lethal violence. The cooperative eye hypothesis predicts that human hyper-cooperation^17^ is at least partially facilitated by the presence of conspicuous white sclera^9^. Human white sclera conspicuously signal iridal direction to conspecifics which may be important in the establishment of joint attentional states^9^. The extent of allomaternal care has been found to best predict variation in proactive prosociality, suggesting that this behaviour may emerge with cooperative breeding systems^17^. Additionally, alloparental care frequencies have been linked to neural control of facial musculature in primates^37^. Furthermore, common marmoset (*Callithrix jacchus*) dyads have recently been shown to more frequently share direct gaze when working on cooperative tasks^38^. These findings are consistent with our results showing that increased cooperation predicts pale sclera and that the four brightest species’ sclerae across our sample are found among humans and cooperative breeders: common marmosets (*Callithrix jacchus*), Goeldi’s marmosets (*Callimico goeldii*), humans, and cotton-top tamarins (*Saguinus oedipus*).

However, the evidence that sclera is communicative outside of humans is sparse and subject to challenges, including small scleral sizes in some species^3,5,6^, as noted above. It may be, then, that white sclera are by-products associated with self-domestication processes^11^, though there has not yet been a comparative phylogenetic investigation into this topic to our knowledge^15^. The self-domestication hypothesis predicts that selection for social tolerance, and against aggression, may generate prosocial behaviour as a correlated by-product^11,13,14^. Therefore, our finding that social tolerance, in addition to proactive prosociality, significantly predicts scleral brightness is consistent with the self-domestication perspective. We therefore cannot statistically differentiate between self-domestication and cooperative eye explanations at this time. Furthermore, these may not be mutually exclusive perspectives. It could be, as an example, that white sclerae in humans originated as a correlated by-product of self-domestication processes and subsequently became the subject of directional selection as neural bases for social cue recognition have developed around this morphology^39^. This may explain why humans appear to uniquely exhibit uniformity in scleral phenotype, i.e., depigmentation, whereas other primates show intra-specific variation^5,6,10,11^. Future research may utilise contrast measures between the iris, sclera^5,6,10^, skin^1,10^ and/or pupil^10,40^ to better evaluate the cooperative eye hypothesis versus the self-domestication hypothesis, which would not require ocular contrast values, in a phylogenetically weighted study. Existing studies that do not utilise modern phylogenetic techniques do not find support for the cooperative eye or gaze camouflage hypotheses^5,6^, potentially rendering self-domestication a better explanation. However, it must be acknowledged that the precise drivers of domestication syndrome are poorly understood^15^ and that it is unlikely any single trait such as scleral depigmentation would be strongly indicative of domestication in the absence of other proposed neural-crest derived traits such as fur depigmentation or cranio-facial anatomy^12,14,15^. An analysis containing additional morphologies would go beyond the scope of this paper but is important, going forwards, to more concretely understand the morphological co-emergents of domestication in primates.

Our results may provide potential drivers of scleral morphologies and show that differences in scleral morphologies may be associated with differences in species’ social behaviour. Recently, an investigation using iridoscleral ratios found humans, bonobos and chimpanzees to have comparable, not distinct, gaze conspicuousness despite large differences in scleral pigmentation^5^. This methodology, however, does not account for physical properties of visible light that may bias the naturalistic perception of shade^6,7,10^, a phenomenon that has recently been shown to influence the successful recognition of an averted gaze, even between primate species^7^. Furthermore, another recent study suggested that variation in primate external eye morphology may be due to genetic drift^6^. Our results contest these findings by showing that differences in species’ social behaviours may be associated with divergent scleral morphologies.

The sample sizes for the behavioural measures of proactive prosociality and social tolerance may be considered small (n = 15), however both represent the high amount of labour required to experimentally extract these data^17^. The experimental methodology used to determine proactive prosociality and social tolerance values^17^ benefits from high environmental control, from animals being allowed to act within their social groups and from control sessions to ensure the functional meaning of the food lever be understood. Although, this method is likewise constrained by the common limitation of food sharing experiments to be artificial and highly context specific. As such, the degree to which these experimentally derived values can be applied as proxies for prosociality or tolerance outside of the context of food sharing/competition is not entirely clear. However, the behavioural values obtained may enable a deeper analysis than the use of common sociality proxies such as social group size. For example, many species, such as humans, chimpanzees and bonobos live in large social groups^3,17^ but yet differ widely in social behaviours such as reactive aggressiveness^13^ or cooperativeness^11^. The use of species-level behavioural data is therefore appropriate, though the smaller sample size (and the use of different species within datasets) does exclude the possibility of multiple regression techniques. Likewise, the use of conspecific lethal violence data^41^ acts as a useful indicator of a species’ aggressiveness. However, the original compilation method did not separate between inter- and intra-group aggression, infanticide or maternal abandonment, behaviours that likely have different neural bases^13^ and may exert different influences on ocular morphology. This may underlie the weaker model fit in the conspecific lethal violence regression.

A limitation relates to the lighting properties of online photographs. Quantitative analyses of colour from digital photographs often require photographs to be taken in controlled settings and/or with the use of a colour standard for calibration^42^. This is not possible when analysing existing, uncalibrated photographs, however, this is less relevant when exclusively analysing brightness rather than the additional hue and saturation values that comprise the full perception of colour (rather than shade alone) from digital sources^40^. The ambient brightness of the photo represents a minor source of unaccounted variation, and although this is mitigated by the use of multiple distinct photos per species (minimum six) with the majority of photos (97.6%) containing both eyes per individual for further average calculations, this remains a limitation to this study. A greater number of photos per species would enable increased average accuracy to mitigate the effect of ambient brightness variation and hence would produce results of greater validity. Likewise, we are unable to account for the potential influence of intra-specific variation in primate scleral pigmentation, although it is not yet known how widely species of non-human primate vary intra-specifically in scleral pigment phenotype. Here, we follow a previously established quantitative methodology^5,6,10,40^ which is preferable to qualitative alternatives of describing shade^4^. Furthermore, this approach, rather than a laboratory-based analysis, relates more closely to the naturalistic perception of ocular morphologies by onlookers since primates will continue to interact with conspecifics across a range of locations and times. Hence, this method enables more ecologically valid testing of the function of sclerae in relation to social interactions. A further minor limitation in using online primate facial photographs is that these images may be biased by the aesthetic preferences of the human photographer and/or uploader, a bias that may particularly influence photographed eye characteristics, as, even when looking at non-human animals, humans often attend to the eyes^43^. In addition, it remains possible that ocular morphologies in addition to the sclera, such as the limbus or temporal wedge^19^, may be captured in analyses due to the presumable variation in the presentation of these features between primate species, which have not been described to our knowledge. However, this is also a limitation common to every previous study concerning divergent ocular morphologies in primates^1–6,10^.

In sum, we provide the first quantitative, comparative analysis of primate scleral pigment. Human white eyes have long been a mystery by comparison to the dark scleral phenotypes observed among many non-human primates. Here, we show that pale scleral colour predicts cooperation whereas dark scleral colour predicts reduced cooperation and increased lethal aggression in extant primates. This refutes the recent notion that divergent eye morphologies can be explained primarily by genetic drift. Results are consistent with the self-domestication, cooperative eye and gaze camouflage hypotheses of eye-behaviour co-evolution.

## Methods

We obtained species-level scores of proactive prosociality and social tolerance across 15 primate species from Burkart et al.^17^. Full methodological details can be found with the original paper. In brief, the authors used a group service apparatus^44^ to measure proactive prosociality and social tolerance. Captive non-human primates were habituated to the apparatus and taught the function of an accessible lever which moved a board containing food into reach. Food was placed on the board in two positions: One where the individual pulling the lever could reach the food themselves and another where the individual pulling the lever could not reach the food themselves, but could make the food accessible to group-mates. The resultant data are comparable between groups and species due to the standardization of this procedure. Proactive prosociality measured how many items of food an individual made available to their groupmates that they themselves could not access. Social tolerance was quantified where the board was in a fixed position with the food accessible and repeatedly replenished (35 times) as it was eaten. Authors then measured the evenness of the distribution of food items within the group to produce social tolerance scores. Lastly, conspecific lethal aggression was scored as the percentage of deaths per species (study populations are taken to be representative of each species) that were attributable to conspecific lethal aggression obtained from published data for 108 extant primate species^41^. There are hence species which are represented in the proactive prosociality and social tolerance models which are not represented in the conspecific lethal violence model and vice versa.

We collected primate facial images from an online google image search completed in February 2021 and which used the common species name and the word “face” as key words. Some chimpanzee and bonobo images were collected from Perea-Garcia et al.^5^ and the Royal Burgers’ Zoo, Netherlands. Human images are public domain and were collected from the Pexels.com database using the key words “man”, “woman”, “face” and “eyes”. We then analysed scleral brightness using ImageJ 1.x^45^. Images were selected from this search to ensure the resolution of the external eye was of sufficient quality, that the eyes were unobscured by other objects and that there was no apparent photo manipulation present. Images were converted to greyscale such that the value of each pixel varied from 0 (black) to 255 (white) with intermediate scores being corresponding shades of grey. We then extracted scleral brightness values following existing techniques^5,6,10,40^. We collected values from within a rectangular selection area placed on the visible sclera for each eye per photo. A minimum of six photos were collected per species, although it is not known whether these represent six distinct individuals. We then calculated the median pixel value per selection area (due to the potential for outliers such as poor lighting, camera quality or light reflections) and the mean value per individual (i.e., mean of both eyes/selection areas), and subsequently the mean scleral brightness value per species. Of the 944 total facial photos, 21 (2.2%) presented with a cranial angle from which only 1 eye was visible. In these few cases, the one accessible eye was taken to be representative of the scleral brightness of that individual. Species which were documented by Gomez et al.^41^, but for which insufficient number or quality of facial photos was available from online materials or which were not uniquely represented in the GenBank taxonomy^46^ were not included in analyses but are listed (Supplementary Table S1). No live animals, only pre-existing photographs, were used during this study.

**Figure 5:**
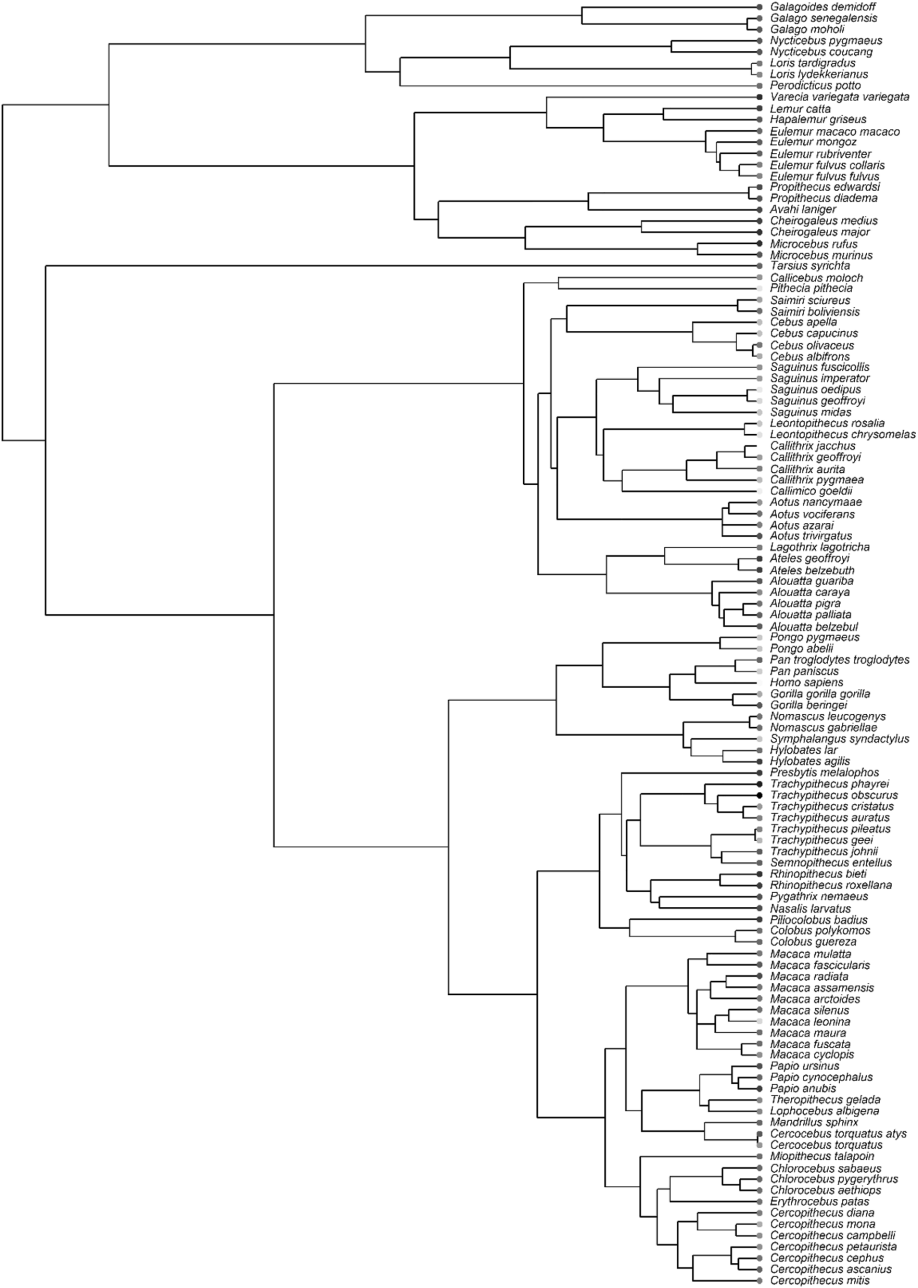
Primate phylogeny with brightness indicator. This shows the phylogenetic relationships between the species used and indicates their scleral brightness. Shade information is continuous.

Phylogenetic least squares analysis (PGLS) was completed in R version 4.0.3.^47^ using the ‘caper’ package version 1.0.1.^48^. A consensus phylogeny (Fig. 5) with branch lengths proportional to time (i.e., a chronogram) was generated and pruned for use from 10ktrees.com version 3^49^ and used the GenBank taxonomy^46^. Variables were log transformed where residuals were non-normally distributed or to improve linearity. Where a variable contained zero-values, it was square root transformed as this does not require the input of an arbitrary constant which influences goodness-of-fit^50^. Diagnostic plots were generated using the ‘plot.pgls’ function^48^ and visually inspected to ensure model assumptions were met. Normality was also determined using Shapiro–Wilk tests on model residuals. The significance value (i.e., alpha) is placed at 0.05. We estimated phylogenetic signal in PGLS analyses using Pagel’s λ, which indicates the degree to which the co-variance in model residuals is proportional to shared evolutionary history between species, assuming a Brownian motion model of evolutionary change over time^51,52^. λ varies from 0 to 1, where 0 indicates that species are independent of one another and 1 the maximum level of phylogenetic signal, i.e., that co-variances are directly proportional to shared evolutionary history^51,52^. Estimates of the maximum likelihood of lambda were subject to wide confidence intervals, a limitation increasingly common with reduced sample sizes^53^ (likelihood profiles: Supplementary Figs. S1–S3). For this reason, and to present a full picture of results, two result statements are provided per statistical test: one where lambda is assumed to equal its maximum likelihood (λ_ML_) and one where lambda is taken to equal 1 (the strictest phylogenetic control). The latter approach is highly conservative as higher phylogenetic control typically reduces the significance of independent variables^54^.

## Supplementary Information

Supplementary Information.

## Data Availability

All data are available at the following repository (https://osf.io/5ac68/) alongside links to the images used. The images are linked but cannot be directly distributed due for copywrite reasons.
